# Viruses of protozoan parasites and viral therapy: Is the time now right?

**DOI:** 10.1186/s12985-020-01410-1

**Published:** 2020-09-29

**Authors:** Paul Barrow, Jean Claude Dujardin, Nicolas Fasel, Alex D. Greenwood, Klaus Osterrieder, George Lomonossoff, Pier Luigi Fiori, Robert Atterbury, Matteo Rossi, Marco Lalle

**Affiliations:** 1 School of Veterinary Medicine and Science, University of Nottingham, Sutton Bonington, Loughborough, Leicestershire, LE12 5RD UK; 2 Molecular Parasitology Unit, Department of Biomedical Sciences, Institute of Tropical Medicine, Nationalestraat, 155, 2000 Antwerpen, Belgium; 3 Department of Biochemistry, Faculty of Biology and Medicine, University of Lausanne, Ch. des Boveresses 155, 1066 Epalinges, Switzerland; 4 Department of Wildlife Diseases, Leibniz Institute for Zoo and Wildlife Research, Berlin, Germany; 5 Department of Veterinary Medicine, Freie Universität Berlin, Berlin, Germany; 6 Institut für Virologie, Robert Von Ostertag-Haus - Zentrum Fuer Infektionsmedizin, Robert von Ostertag-Str. 7-13, 14163 Berlin, Germany; 7 Department of Biological Chemistry, John Innes Centre, Norwich Research Park, Norwich, NR4 7UH UK; 8 Dipartimento Di Scienze Biomedice, Universita Degli Studi Di Sassari, Sardinia, Italy; 9 Unit of Foodborne and Neglected Parasitic Diseases, European Union Reference Laboratory for Parasites, Department of Infectious Diseases, Istituto Superiore Di Sanità, viale Regina Elena 299, 00186 Rome, Italy; 10 Department of Infectious Diseases and Public Health, Jockey Club College of Veterinary Medicine and Life Sciences, 31 To Yuen Street, Kowloon, Hong Kong

**Keywords:** Parasite, Virus, dsRNA, Therapy, Virus-like particles

## Abstract

Infections caused by protozoan parasites burden the world with huge costs in terms of human and animal health. Most parasitic diseases caused by protozoans are neglected, particularly those associated with poverty and tropical countries, but the paucity of drug treatments and vaccines combined with increasing problems of drug resistance are becoming major concerns for their control and eradication. In this climate, the discovery/repurposing of new drugs and increasing effort in vaccine development should be supplemented with an exploration of new alternative/synergic treatment strategies. Viruses, either native or engineered, have been employed successfully as highly effective and selective therapeutic approaches to treat cancer (oncolytic viruses) and antibiotic-resistant bacterial diseases (phage therapy). Increasing evidence is accumulating that many protozoan, but also helminth, parasites harbour a range of different classes of viruses that are mostly absent from humans. Although some of these viruses appear to have no effect on their parasite hosts, others either have a clear direct negative impact on the parasite or may, in fact, contribute to the virulence of parasites for humans. This review will focus mainly on the viruses identified in protozoan parasites that are of medical importance. Inspired and informed by the experience gained from the application of oncolytic virus- and phage-therapy, rationally-driven strategies to employ these viruses successfully against parasitic diseases will be presented and discussed in the light of the current knowledge of the virus biology and the complex interplay between the viruses, the parasite hosts and the human host. We also highlight knowledge gaps that should be addressed to advance the potential of virotherapy against parasitic diseases.

## Background

According to CDC, “a parasite is an organism that lives on or in a host organism and gets its food from or at the expense of its host*”* (https://www.cdc.gov/parasites/about.html)*,* and for the purposes of this review the term parasite will exclusively refer to eukaryotic organisms. Protozoa, together with helminths, represent the main cause of parasitic disease in humans in addition to livestock and companion animals [1, 2]. Despite the great advances in modern medicine, parasitic infections continue to burden the world with huge costs in terms of human and animal health, and national economies directly and indirectly [3]. The prevalence of the major human protozoan parasitic diseases (PPDs) is estimated to be ca. 790 million individual cases, with a yearly death toll of 810,000 and 82.4 million Disability Adjusted Life Years (DALY) [4]. Additional indirect negative effects on human health can result from zoonotic parasitic infections [5]. Most PPDs are widely regarded as poverty-related and as neglected tropical diseases, largely ignored for many years by health authorities, pharmaceutical companies and the media. It is perhaps not surprising that with such a low profile, drugs, vaccines, surveillance and control tools are lacking for most of these diseases and disease control remains problematic [6–11]. New approaches to chemotherapy are needed urgently, involving combination therapies and other strategies, in addition to cheaper, less toxic drugs. A paradigm shift is also required in the development of chemotherapeutic drugs that; (1) target key parasite-specific metabolic pathways; (2) do not lead to the development of resistance or increase virulence as a result of the parasite genomic and metabolic plasticity; (3) are active against dormant parasite stages (e.g. *Plasmodium vivax* hypnozoites, *Toxoplasma gondii* cerebral tissue cysts, etc.); and (4) are applicable for asymptomatic cases that act as reservoirs. Vaccine development for PPDs remains a serious problem for several reasons. Host–pathogen interactions are generally poorly understood and, in the case of vector-borne diseases and protozoan parasites, they are complicated by the immune response to insect components introduced with the parasite [12]. Protozoan parasites are highly efficient at immune escape, with mechanisms including (1) antigenic variation, as occurs with *Plasmodium*, *Giardia* and the African Trypanosomes [13], (2) dormancy and seclusion in safe target tissues, as reported for *Plasmodium*, *Toxoplasma* and *Leishmania* [14], (3) subversion of host defences reported for *Leishmania* [15, 16], (4) capping of host immunoglobulins with proteinases as occurs with *Trichomonas* [17], (5) production of anti-apoptotic factors by *Cryptospodium* [18], or (6) down-regulation of host immunity [19]. The use of antimicrobial peptides, aptamers, nanoparticles, extracellular vesicles and natural antimicrobials, including probiotics, are currently under investigation to evaluate their feasibility as alternative therapeutic agents, alone or in combination with chemotherapy [20–23].

To the same end, speculation has begun on the feasibility of using viruses, in particular those infecting parasites [24] for controlling PPDs. The precedent is that bacterial viruses (bacteriophages) and human viruses with lytic activity for malignant cells (oncolytic viruses), have already been used successfully against antibiotic-resistant bacteria and cancers, respectively.

The first clear evidence of viral endosymbionts in parasites was the discovery by transmission electron microscopy (TEM) of virus-like particles (VLPs) in parasites such as the protozoans *Entamoeba histolytica* and *Leishmania hertigi* (currently *Paraleishmania hertigi*) [25, 26] and the platyhelminth *Diplectanum aequans,* a parasite of fishes [27]. Since then, VLPs and true viruses have been documented in a variety of protozoan and helminth parasites that in turn parasitize humans, animals, plants [28–35], with many more expected to be discovered in the near future due to the extensive application of high-throughput sequence technologies [36].

A complete review of the viruses of protozoan parasites is beyond the scope or intention of this review. Rather, we will focus mainly on the protozoan parasites *Trichomonas vaginalis*, *Leishmania* spp., *Giardia duodenalis* and *Cryptosporidium spp*., and their viral endosymbionts, as model systems to present and discuss the potential for exploiting their use as native or manipulated viruses to treat human parasitic diseases together with the challenges associated with their application.

## Main text

### Viral endosymbionts of protozoan parasites

**Fig. 1:**
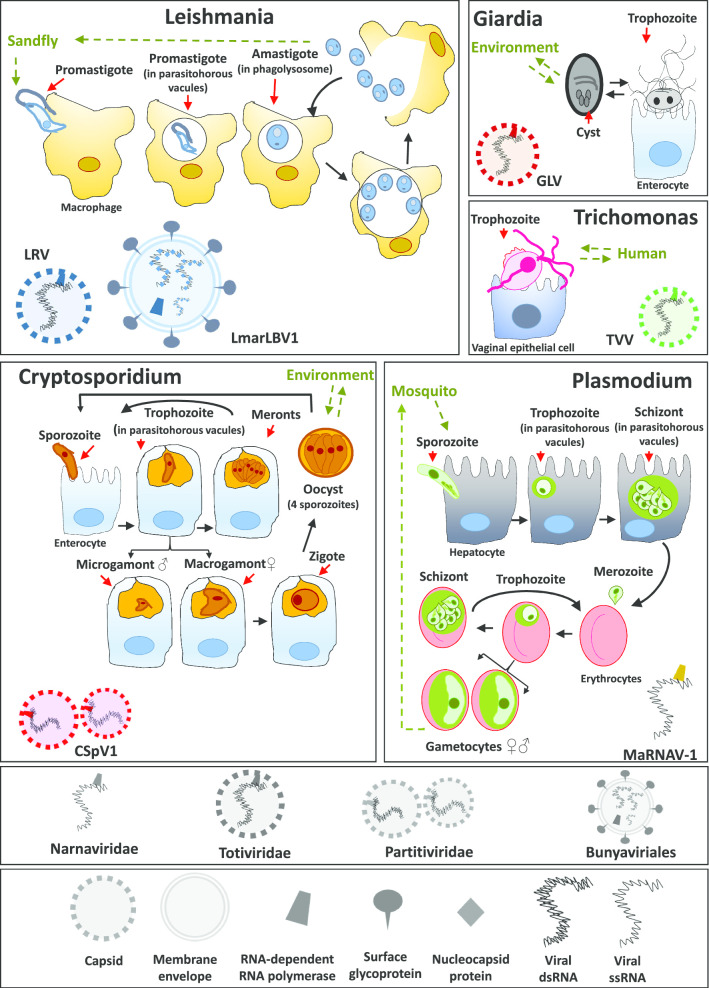
Protozoan parasites and their viral endosymbionts. The life stages in the human (or mammalian) host of Leishmania spp., Giardia duodenalis, *Trichomonas vaginalis*, Cryptosporidium spp, and Plasmodium spp, are depicted together with a graphical representation of the corresponding viral endosymbionts (see also Table 1). Leishmania. Promastigotes, injected in the mammalian host during a sandfly blood-meal, are taken up by macrophages in the dermis and quickly surrounded by a parasitophorous vacuole (PV). Promastigotes differentiate into non-motile amastigotes and proliferate inside the phagolysosome. Following lysis of infected macrophages, free amastigotes can infect neighbouring macrophages. Infected macrophages and/or free amastigotes may then be ingested by sandflies. Giardia. The cyst ingested by the mammalian host releases trophozoites that multiply by binary fission and colonize the upper part of the small intestine by adhering to the enterocyte surface. Following specific stimuli, trophozoites differentiate back to cysts that are released into the environment in the stool. Trichomonas. Trophozoites are transmitted sexually between humans where, by binary fission, they colonize the lower genital tract of females and the urethra and prostate of males, No cyst form is known. Cryptosporidium. Oocysts ingested by the mammalian host release sporozoites that invade the epithelial cells of the small intestine, form an extra-cytoplasmic yet intra-cellular PV and differentiate into trophozoites. Asexual multiplication by schizogony generates meronts that can infect new enterocytes. Eventually trophozoites differentiate into female macrogamonts and male microgamonts. After fertilization, the zygote develops into an oocyst that will exit the host through the faeces. Plasmodium. Sporozoites injected in the mammalian host during a mosquito blood-meal, invade the hepatocytes, differentiate into trophozoites within a PV and multiply asexually by schizogony giving rise to schizont containing many merozoites (hepatic cycle). Hepatic merozoites then invade erythrocytes (RBC) and the schizogonic multiplication occurs with newly released merozoites capable of infecting new RBC. Trophozoites in RBC can eventually differentiate in male and female gametocytes that will reach mosquitoes during a blood-meal

Based on the categorization of virus families by their genetic material, mode of replication and structural properties, the most extensively characterized viral endosymbionts of protozoan parasites of medical relevance are small, non-enveloped, double-stranded (ds) RNA viruses of the family Totiviridae [37], with other viruses being described including members of the Partitiviridae (non-enveloped dsRNA, bipartite), Narnaviridae (uncapsidated, single-stranded positive, ssRNA (+) and, monopartite Bunyaviridae (enveloped, single-stranded negative, ssRNA (−), tripartite) (Table 1 and Fig. 1).

**Table 1:** Summary of main properties of viruses known to infect protozoan parasites of medical relevance

Pathogen	Pathogen species	Virus (family/genus)	Genome	Genome size	Virion (shape/diameter)	Main ORFs	References
*Leishmania*	*L. guyanensis* *L. brasiliensis* *L. shawi*	LRV1 (Totiviridae/Leishmaniavirus)	Monopartite, linear dsRNA	5.3 Kb	Icosahedral/~ 40 nm	CP and CP/RdRp	[80, 81, 164]
	*L. major* *L. aethiopica* *L. infantum* *L. tropica*	LRV2 (Totiviridae/Leishmaniavirus)	Monopartite, linear dsRNA	5.2 Kb	Icosahedral/~ 40 nm	CP and CP/RdRp	[82, 83]
	*L. martiniquensis*	LmarLBV1 (unassigned /Leishbunyaviruses)	Tripartite, linear ssRNA(-)	6.1 Kb (segment L) 1.2 Kb (segment M); 0.7 Kb (segment S)	Enveloped, spherical/~ 100 nm	ORF L; ORF M and ORF S	[99]
*Giardia*	*G. duodenalis*	GLV (Totiviridae/Giardiavirus)	Monopartite, linear dsRNA	6.3 Kb	Icosahedral/ ~ 48 nm	CP and CP/RdRp (2 partially overlapping ORFS)	[63]
*Trichomonas*	*T. vaginalis*	TVV (1–4) (Totiviridae/Trichomonasvirus)	Monopartite, linear dsRNA	4.5–5 Kb	Icosahedral/~ 33 nm	CP and CP/RdRp (2 partially overlapping)	[42, 43]
*Cryptosporidium*	*C. parvum* *C. hominis* *C. felis* *C. meleagridis*	CSpV1 (Partitiviridae/Cryspovirus)	Bi-segmented, linear dsRNA	1.8 Kb (dsRNA1) 1.4 Kb (dsRNA2)	Icosahedral ~ 31 nm	RdRp (dsRNA1) and CP (dsRNA2)	[101, 102]
*Plasmodium*	*P. vivax*	MaRNAV-1 (unassigned/narna-like virus)	Bi-segmented, linear ssRNA (+)	2.9 Kb (segment I) 2.6 Kbp (segment II)	No true virion	RdRp (segment I) and CP (segement II)	[33]

Within the Totiviridae family, 5 genera, *Giardiavirus, Trichomonasvirus, Leishmaniavirus, Totivirus,* and *Victorivirus,* are currently recognised which share common characteristics [37]. Their genomes are linear uncapped dsRNA encoding for two partially overlapping proteins; the capsid protein (CP) and the RNA-dependent RNA polymerase (RdRp). The RdRp is generally expressed as a CP/RdRp fusion protein by means of a − 1 or, more rarely, a + 1/− 2 ribosomal frameshift or by ribosomal hopping [37]. The viral genome is never found free in the protozoan cell and the positive strand viral transcript is synthesized within the viral particle by CP/RdRp and translocated to the cell cytoplasm to be translated [37]. The virions (with a median size of 40 nm) are icosahedral, composed of 120 copies of capsid protein with a "T = 2" symmetry (alternatively defined as T = 1 icosahedral lattice arranged as 60 asymmetric homodimers) [37, 38].

#### Trichomonas vaginalis and Trichomonasvirus

The flagellated protozoon *Trichomonas vaginalis* is responsible for 170 million cases/year of trichomoniasis, the most common non-viral sexually transmitted infection worldwide [39]. Although asymptomatic in males, symptoms of trichomoniasis in women may vary from asymptomatic to severe vaginitis, eventually with pregnancy and postpartum complications [40]. The *Trichomonas vaginalis* virus, TVV, belongs to the genus *Trichomonasvirus* and was the first virus from a protozoan parasite to be described and characterised in the 1980s, and the first for which the full-length genome sequence was reported [41, 42]. Four main phylogenetically distinct viral species, 1 to 4, have been described, with TVV1 closer to TVV2 and TVV3 to TVV4 [43]. Diversity exists within each TVV species, with, for example, translation of the CP/RdRp fusion protein of TVV1 involving a -2 ribosomal frameshift unlike TVV2-4 [44, 45]. Co-infection of a single *T. vaginalis* with different TVV strains has been reported [43]. The TVV infection rate among *T. vaginalis* strains from different geographic origins ranges from 40 to 100%, with TVV1 being the most commonly detected [46]. TVV seems to be transmitted only vertically, although some studies suggest a correlation between specific genetic polymorphisms and the entry and multiplication of TVV [42, 44, 47]. The presence of TVV influences negatively the growth rate of *T. vaginalis *in vitro if compared to uninfected protozoan isolates [48] and there is also evidence for lytic effects of TVV on *T. vaginalis* [49]. Almost 50 proteins are expressed differentially between TVV-infected and uninfected isolates, including metabolic enzymes, heat shock proteins (down-regulated in TVV-positive strains), and ribosomal proteins (up-regulated in TVV-positive strains) [50]. Indeed, infection with TVV increases both cytoplasmic and surface expression of the p270 protein, the major immunogenic protein of *T. vaginalis*, in a phosphorylation-dependent fashion [51]. Similarly, TVV infection can modulate the quantitative and qualitative expression of the protozoan cysteine proteases [52]. Since cysteine proteases are involved in modulating *T. vaginalis* cyto-adherence to human host cells and in degradation of basement membrane, human cellular molecules, and secretory IgAs, the viral endosymbiont seems to influence and modulate protozoan virulence [52]. A correlation between TVV symbiosis with *T. vaginalis* isolates and the severity of clinical symptoms of trichomoniasis in humans is emerging; while different papers report a positive association between TVV infection and the exacerbation of trichomoniasis symptoms, other authors have shown the absence of any correlation [53]. However, *T. vaginalis* and its virus appear to have a clear role in the subversion of the innate immune response and inflammation in the human host [54, 55]. Although TVV is unable to infect and replicate in human cells [56], its presence can modulate the pro-inflammatory response in the human host, amplifying the innate response, and thus exacerbating clinical symptoms and the severity of disease. The viral dsRNA and TVV particles can be sensed by receptors exposed on vaginal cells, triggering NF-κB activation via endosomal TLR3/TRIF-dependent pathways and leading to expression of Interferon Type 1 genes [54]. This release of viral dsRNA may be favoured by the presence of wide channels in the virion [45]. Although the percentage of clinical *T. vaginalis* isolates resistant to 5-nitroimidazole treatment is increasing [57], and although a correlation with the presence of TVV has been postulated, this is still debated and poorly understood [53, 58, 59]. Paradoxically, in the case of infection caused by *T. vaginalis* carrying TVV, failure of anti-protozoan therapy with metronidazole in order to prevent preterm delivery in pregnant women results in an exacerbated inflammatory response explained by the increased release of virions and dsRNA as a result of parasite killing [45, 60]. The co-existence of dsRNA virus may also disturb the equilibrium of the mucosal microbiome, contributing to its modification in the vagina; infection with TVV*-*positive *T. vaginalis* isolates indeed promotes vaginal colonization by pathogenic bacteria associated with bacterial vaginosis while decreasing the adherence to the vaginal epithelium of the major vaginal microflora that dominate during eubiosis [42, 54].

#### *Giardia duodenalis* and *Giardiavirus*

The flagellate protozoan parasite *G. duodenalis* (syn. *G. lamblia, G. intestinalis*) infects the upper intestine of humans and other mammals causing giardiasis, a zoonotic diarrhoeal disease. The parasite has a global distribution with 250–300 million symptomatic human infections reported annually, and its impact is more pronounced in the developing world and under poor socioeconomic conditions [61]. Although self-limiting, *Giardia* infections can become chronic and predispose individuals to other chronic gastrointestinal disorders such as irritable bowel syndrome (IBS) [62]. Soon after the discovery of TVV, the *Giardia lamblia virus* (GLV) was described [63] which is included in the genus *Giardiavirus* together with the virus isolated from the fungus *Gigaspora margarita* [37]. The complete viral genome from three isolates is available [64, 65], providing evidence of minimal sequence variability. GLV shows some unique characteristics among the Totiviridae: (1) CP translation is driven by an unusual Internal Ribosomal Entry Site (IRES), spanning the 5′-UTR (untranslated region) and the initial portion of the CP coding region [66]; (2) the GLV particle is the largest, most robust and thermo-stable among Totiviridae allowing extracellular horizontal transmission of the virus [67]. Susceptibility of *G. duodenalis* to GLV infection has been demonstrated both by parasite transfection with GLV ssRNA and incubation with purified GLV virions, with infected parasites being maintained stably over time [68, 69]. GLV virus entry is thought to occur via receptor-mediated endocytosis since it can be prevented by specific blocking agents [70] and not all *Giardia* isolates are susceptible to GLV infection [69]. GLV was shown to be unable to infect other species of protozoan parasites [59] or to induce cytopathic effects in kidney or intestinal mammalian cell lines [71, 72]. GLV has been detected, by PCR or gel electrophoresis, in more than 30% of *G. duodenalis* isolates belonging to different genetic groups (assemblages), both host-specific and zoonotic [73–76]. Different cytopathic effects, including growth arrest and parasite lysis, have been observed on naïve *G. duodenalis* isolates when first infected with different GLVs purified from naturally infected *G. duodenalis* isolates [63, 73]. However, the reported effects could be explained by differences in either (1) the amount of virus administered, (2) the properties, such as replication efficiency, of each virus isolate or (3) variation in parasite susceptibility to viral infection [73]. No robust evidence associates GLV infection with resistance to metronidazole, the first-line antigiardial drug [77].

#### *Leishmania* and *Leishmaniaviruses*

Different species of the trypanosomatid protozoan *Leishmania* are the causative agents of cutaneous (CL), visceral (VL) or mucocutaneous (MCL) and disseminated (DCL) leishmaniasis, afflicting > 12 million people worldwide, with 1.2 million new cases/year. The parasites are transmitted by phlebotomine sand flies [78, 79]. Within *Leishmania* spp., the Leishmania RNA viruses (LRVs) are isolated mainly from the *Leishmania Viannia (V.)* subgenus from South America, and designated LRV1 [80, 81], whereas from the old-world *Leishmania* subgenus (i.e. *L. aethiopica*, *L. major* and *L. tropica*) were named LRV2 [82, 83]. LRV1 and LRV2 genomes differ slightly [84] with limited sequence homology of ca. 40% at the protein level. LRV transmission from one parasite to another must occur vertically during cell proliferation, as there is no evidence of horizontal transmission. Nevertheless, a recent study suggested that LRV particles are also present in parasite exosomes which might implicate horizontal transmission [85]. Although a systematic review with meta-analysis reported a possible estimated prevalence of 26%, this value is difficult to be estimated since *Leishmania* parasites have the tendency to lose their LRVs endosymbiont in culture [86]. Viral loss possibly relates to a burden for parasite replication that in natural conditions is counterbalanced by the selective advantage provided by LRV within the sand fly vector, in the mammalian host or in both. Supporting this hypothesis LRVs are found mainly in *Leishmania* spp. equipped with RNA interference machinery (the system allowing the cell to actively recognize and degrade non-self dsRNA, likely to be mainly of viral origin) suggesting the importance of the parasite’s anti-viral defense in finely balancing viral replication rate [87]. Differently from TVV, the presence of LRV does not affect parasite growth and there is no evidence of modulation of parasite gene expression [88]. Despite LRV has been isolated from active and healing lesions and scars [89–91], thus far no experimental system has allowed long-term maintenance of LRV in *Leishmania* following experimental transfer (N. Fasel personal communication). The presence of LRV is reported to significantly promote disease relapses in humans infected with *L. braziliensis*, *L. guyanensis* or *L. naiffi* and receiving antimony or pentamidine treatment [92]*.* MCL, characterized by the dissemination of the infection to secondary sites with a high inflammatory component, has been associated with the presence of LRV in the cytoplasm of the parasites [93]. Another striking effect of LRV is its impact on treatment failure, while the viral dsRNA is also suspected to participate in difficulties in treating HIV/*L. braziliensis*/LRV co-infected patients [94]. How LRV impacts drug resistance and relapse is not yet known but thanks to the development of different animal models a number of mechanisms underlying disease progression have been identified. Similar to TVV, the pathology-exacerbating role of LRV relies on the subversion of the host innate immune response, as was shown for *L. guyanensis* or *L. aethiopica* infection, where the virus exacerbates the disease by inducing hyper-inflammation and increasing the parasite burden as well as lesion size [95, 96]. These phenotypes depend on Toll-like receptor (TLR) 3-mediated recognition of viral dsRNA, which leads to the production of Type 1 Interferon (IFN-I), and pro-inflammatory cytokines and chemokines [97]. Furthermore, LRV promotes the survival of the mammalian cells infected by *Leishmania* by phosphorylating the AKT1 pro-survival kinase, and favors parasite dissemination via the induction of IL-17 production [93, 98].

Noteworthy, a tri-segmented linear negative-stranded RNA virus (termed, LmarLBV1) with characteristics of leishbunyaviruses (an unassigned bunyaviridae-like group of viruses) was recently discovered in *Leishmania martiniquensis*, a protozoan transmitted by biting midges and responsible for severe visceral disease in humans [99]. The presence of LmarLBV1 slightly increases the in vitro infectivity of *L. martiniquensis* on primary murine macrophages [99]. Compared to Totiviridae, Bunyaviridae have enveloped and spherical virions of around 100 nm suggesting that they could be shed by *L. martiniquensis* cells, allowing the virus to interact with the immune system of the human host [99].

#### Other viruses of protozoan parasites of medical relevance

Viruses have been found in other protozoan parasites responsible for serious illness in humans, including the apicomplexan *Cryptosporidium* spp*.* and *Plasmodium vivax.* However, information on the effect of viral infection on these parasites is fragmentary.

Different species of *Cryptosporidium* (i.e. *C. parvum, C. hominis, C felis and C. meleagridis*) are pathogenic for humans and other vertebrates, and are responsible for cryptosporidiosis, a severe diarrhoeal disease causing death in young children especially in developing countries [100]. A bi-segmented dsRNA, termed *Cryspovirus,* of the family Partitiviridae and commonly associated with plants and fungi, was originally detected in *C. parvum* isolates, and later in other *Cryptosporidium* spp.[101, 102]. The dsRNA segments 1 (1.8 Kb) and 2 (1.4 Kb), likely uncapped and not polyadenylated, encode the RdRp and CP, respectively. The isometric virion is composed of 120 subunits (T = 1) of the 37 kDa CP, the smallest capsid protein known among Partitiviridae with each viral genome segment encapsidated separately [102]. As with other partitiviruses, CspV1 is thought to be transmitted intracellularly, being present in the environmentally resistant oocyst stage, although recent observation suggests that CspV1 is also released into the medium early in the parasite infection of the human host cell [103]. Based on dsRNA2 sequence comparison, a greater divergence exists between CSpV1 and viruses from *C. hominis*, *C. felis* and *C. meleagridis*, suggesting that the virus might have a certain degree of host-specificity and, therefore, the existence of more than one species in the genus *Cryspovirus* [104]. Infection by CSpV1 is commonly detected in field and clinical isolates of *C. parvum* [105]. A correlation between CSpV1 dsRNA2 levels and *C. parvum* fecundity has been reported in culture models, where higher level of the viral symbiont are associated with greater parasite multiplication [106]. However, no information on infection in an animal model is available.

Six *Plasmodium* spp. (*P. falciparum, P. vivax, P. malariae, P. ovale curtisi, P. ovale wallikeri, and P. knowlesi*) infect humans, transmitted by the bite of an infected female *Anopheles* mosquito, and are responsible for malaria, the most important PPD affecting humans, with an incidence of more than 200 million case/year and 400 thousand deaths/year [107]. A bi-segmented narna-like ssRNA (+) virus has been very recently isolated only from *P. vivax* and named Matryoshka (after the Russian dolls) RNA virus 1 (MaRNAV-1) [33], showing high homology to the monopartite, linear, positive sense (ss)RNA narnaviruses found in fungi, plants and other protists. The trypanosomatid *Leptomonas seymouri*, transmitted by the sandfly vector, as is *Leishmania donovani*, can also harbour a symbiotic narna-like virus (LepsyNLV1) [108]. It is noteworthy that severe cases of visceral leishmaniasis in India have been associated to co-infection with by *L. donovani* and a narna-like positive *L. seymouri* [109].

### What can we learn from approved viral therapies; bacterial viruses (bacteriophage) and oncolytic viruses?

Viruses are already in use in medicine, the two best-known examples being bacterial viruses (bacteriophages, phages) to treat bacterial infections and oncolytic viruses (OVs) in the treatment of cancer [110].

The specificity of bacteriophages, absence of adverse effects on the normal bacterial flora (unlike antibiotics), their greater efficacy than some antibiotics [111] and little evidence of them causing harm on administration are major advantages for their application. However, bacteriophages as a generic group are not yet considered to have a qualified presumption of safety (QPS) by the EU [112] although they have been used several times in the US for untreatable infections [113].

Their successful use to control and prevent a variety of bacterial infections is based on their lytic activity but is conditional on their use under carefully controlled conditions. These include phage preparation according to good manufacturing practices, demonstration of phage efficacy and safety in randomized controlled trials and marketing authorization. Indeed, the evidence for their efficacy is a combination of (1) a huge number of individual treatments in Eastern European countries involving different bacterial infections, (2) highly controlled experimental infections using animal models of enteritis, septicaemia, superficial burns and other types of infections, and (3) much earlier trials carried out in the 1920s–1930s some of which, admittedly, were criticised for their poor scientific quality [114, 115]. In the fight against antibiotic resistance (AMR), phages specific for the sex pili produced by self-transmissible AMR plasmids can be employed for the replacement of an AMR strain by an antibiotic-sensitive derivative in a bacterial population [116]. This is a good example of how the complex evolutionary relationship between a virus and its host (i.e. bacteria, bacteriophage and self-transmissible plasmids) can be used to our advantage.

It should, however, be noted that some phages are able to transfer bacterial genes by transduction, a factor that must be taken into consideration during the early stage of phage selection. A comprehensive analysis of theoretical advantages of phages over antibiotics, together with some of their limitations, has been reviewed [117, 118] including the degree of synergy with antibiotics. However, phage-bacterial co-evolution is of immediate practical significance to phage therapy in the development of bacterial resistance to the phage during treatment. Several strategies have been used to overcome this issue [117], including: (1) the use of two phages, one of which targets the original bacteria and the second of which targets the phage-resistant mutants that arise in response to the first phage [111], and (2) selecting phages that target surface virulence determinants so that most phage- resistant mutants are attenuated and thus do not produce disease [111]. Interestingly, in the case of the use of phage against the pili produced by AMR bacteria, the selection of phage-resistant mutants is desired. In addition to exploitation of their lytic activity, phages have been also manipulated to deliver toxin genes into pathogens as an alternative to chemotherapy [119], or to deliver a functional CRISPR-Cas system destroying antibiotic resistance plasmids [120, 121]**.** However, despite their great promise, reasons for the slow adoption of phages in human medicine and the paucity of randomised, double blind trials [122–124] can be found, among others, in the difficulties with intellectual property and registration, some of which may be overcome by the use of magistral (personalised) phages [125] for treatment of individual infections.

A variety of human and animal viruses have been tested for their application as oncolytic viruses (OVs) including herpes viruses, pox viruses, the Edmonton strain of Measles virus and, most frequently, adenoviruses since they have few side effects and their genomes are easily manipulated. The ability of viruses to target cancer cells arises from their exploitation of the aberrant signaling pathways and the generally poor antiviral response produced by cancer cells [126].

Many viruses are able to induce lysis of cancer cells in vitro but their effect in vivo appears primarily to involve modification of the micro- and macro-environment of the tumor with the induction of apoptotic pathways and stimulation of innate immunity by TLR activation [127]. Contact between cancer cells and virus triggers a stress response involving reactive oxygen species (ROS) and release of Damage-Associated Molecular Patterns (DAMPs), also initiating an immune response [128]. Although OVs lyse a variety of cell types in vitro*,* the outcomes of their clinical use are enhanced by combination with other treatments, such as immune therapy [129] or general or targeted chemotherapy [130] for a wide range of cancer types [126].

Oncolytic viruses (OVs) have been transformed from the object of laboratory studies [126] to full scale clinical trials with the acceptance of three OVs for full clinical use [126]. A non-pathogenic ECHO virus has been registered for use in several Eastern European countries, an attenuated adenovirus has been registered for use in China and a modified herpes virus (HSV-1) was approved by the FDA and EMA in 2015 and is now used routinely for certain melanomas [126].

OV therapy has a number of advantages over current antitumor therapies. OVs can selectively replicate in tumor cells [127] and can act as vectors for therapeutic and immune-stimulatory genes [132] for specific expression at tumor sites [133]. Some OVs are reported to cross the blood–brain barrier and they can increase the sensitivity of cancer cells to other immune-therapies. Interestingly, and, unlike bacteriophages, resistance to the viruses has not so far been observed. In theory, and similarly to bacteriophages, virus dose in the tumor increases as a result of in situ virus amplification that is different to normal drug pharmacokinetics [127].

The general safety of oncolytic viral therapy has been highlighted by published clinical trials, although some aspects, including off-target effects, unexpected toxic effects as result of the viral genome manipulation, virus mutation, evolution, and recombination, remain a theoretical concern [134]. Other potential limitations relate to the requirement of the viruses to reach every cell in the tumor and to spread between the tumor cells and the occurrence of neutralizing antibodies in the host that could result in a rapid shut down of viral replication. By the incorporation of biological markers in OVs they can also play an integral role of tumor visualization [135].

### Exploitation of viruses of protozoan parasites–scope for therapy

The experience with oncolytic viruses and bacteriophages suggests that parasite viruses may be used in a variety of ways, including via their own lytic activity, possibly synergistically with other approaches to treatment including immunotherapy and chemotherapy, for drug delivery using virus-like particles (VLPs) and also by manipulating their genomes for specific purposes.

#### Application of viruses by their direct parasitopathic activity

To maximize any parasitopathic activity of a viral symbiont, the selectivity of the virus toward the right parasite target must be high. This seems to not be an issue, at least for GLV, TVV and LRV viruses [56, 63]. Moreover, replication of the virus should be maximal at the most appropriate parasite stage infecting the human (or animal) host. For those protozoan parasites with relatively simple life cycles, such has *Giardia*, *Cryptosporidium* and *Trichomonas*, with parasite multiplication occurring predominantly in one site (e.g. gastrointestinal epithelium for *Giardia* and *Cryptosporidium*, vaginal epithelium for *Trichomonas*) within the final human (or animal) host, the endosymbiont virus is likely to replicate maximally where the parasite is also multiplying (e.g. trophozoite for *Giardia* and *Trichomonas*, amastigote for *Leishmania*, merozoite for *Cryptosporidium*) (Fig. 1). The picture is likely to be more complex when different replication stages occur during the parasite life cycle, as for *Leishmania* and *Plasmodium*, for instance, and in more than one host (i.e. intermediate and definitive hosts) in addition to multiple sites (tissues or organs) in the same host (Fig. 1). Thus, viruses that multiply optimally in one phase of the life cycle might, nevertheless, be used profitably for infection control in another. These speculations remain to be tested. The extent to which the viruses might be used alone or synergistically also remains to be assessed.

Such use of wild-type viral symbionts seems, at the moment, to be applicable only to *Giardia* (at least for some parasite isolates) where parasite overload with GLV causes cell growth arrest, and likely cell death [63, 73]. Of course, for this kind of strategy some key aspects must be taken into account, such as: (1) an efficient cell factory for high titre virus production; (2) pharmaceutical grade purification of the viral particles for treatment use, and (3) a successful delivery system for the administration of the viral dose to the patient. Problems associated with human (or animal) immune response to the viral administration must of course be evaluated in advance. It is evident that TVV and LRV might be disadvantaged in this specific issue due to their adverse impact on the human immune system [54, 55, 95].

#### Use of VLPs for chemotherapy

An alternative to using infectious viruses to attack parasites, is their use as VLPs to deliver toxic antiparasitic agents to the parasite host cells. VLPs consist of the assembled viral coat protein subunits that provide empty viral shells which can be loaded with the desired material. The outer surface can also be decorated with targeting moieties, either genetically or chemically. VLPs based on plant viruses have been exploited particularly for this application as they can be produced in large quantities, are stable and can often be readily disassembled and reassembled in vitro to allow the incorporation of drug molecules [136–138].

To date, much of the research on the use of VLPs has concentrated on delivering drugs for anti-cancer therapy [139–142]. However, there has been increasing interest in using VLPs to deliver agricultural pesticides to combat plant-parasitic nematodes [143, 144]. The encapsulation of nematicides within VLPs increases their mobility and persistence, although the effect varies with the nature of the VLP [143, 144]. These recent results indicate that drug delivery via VLPs may be an effective way of treating parasites other than those of plants. Of particular concern in anti-parasitic drug therapy in humans and animals is, for example, the low bioavailability (e.g. poor bioavailability of benzimidazoles) or the off-target effect (e.g. nitroimidazoles also targeting commensal anaerobic bacteria in the intestine) of certain drugs [62, 146] Use of VLP delivery could indeed help to overcome such undesired limitations thus improving treatment quality and increasing drug efficacy. Indeed, the viral particles would be depleted of their nucleic acid content and, in the case of TTV and LRV, this would avoid adverse human host response to the viral dsRNA release [54, 95]. Among the obstacles in production of VLP for therapeutic purposes are, in addition to those for preparation of wild-type virus, the need to express at high yield the sole protein(s) constituting the capsid and guaranteeing the correct assembly of the viral particles, likely in an appropriate heterologous biological system, in addition to efficiently loading the antiparasitic agent.

#### The potential use of the viral symbiont for molecular manipulation of the parasite host

Several technical approaches may be contemplated for the delivery of nucleic acid sequences that will interrupt parasite physiology or kill the parasite. The introduction of antisense sequences to block translation of key genes has been discussed for modulating bacterial physiology but is thought to be less effective and not as easy as originally thought [147], with tRNA processing with external guide sequences as an example [148]. For instance, morpholinos (DNA oligomers modified with methylenemorpholine rings) have been used successfully for transient knock-down of essential genes in *Giardia* and *Cryptosporidium* [149, 150]. Other approaches could be used to target genes directly with key function(s) for parasite survival. More recently, the CRISPR-Cas system has been used increasingly for making targeted mutations being successful also in *Giardia*, *Plasmodium, Trichomonas* and *Leishmania* [151–154]. The system uses guide RNAs to direct a DNA nuclease complex to a target sequence by complementarity with the guide DNA. This has been modified by using dCas9 which is a double nuclease active-site mutant that binds to the DNA target selectively, in this case leading not to cleavage but to inhibition of RNA polymerase initiation and elongation [155]. However, antisense molecules and gene-editing systems do not readily cross cell membranes limiting their use in in vitro systems where techniques, such as electroporation, have been developed to introduce the molecules into cells. VLPs could, indeed, be very useful for their delivery in vivo. An example of such use of the viral symbiont is represented by engineered GLV to express hammerhead ribozymes [156]. The use of catalytic ribozymes [157] to inactivate target genes is a good example but it may be dependent on the relative transcription rates of the genes involved and may also be affected by poor turnover rates under physiological conditions. In *Giardia,* this approach has been successful to knock down specific genes and also provided evidence for the possibility of combining gene silencing by virus-mediated hammerhead ribozymes with chemotherapy [156]. However, this approach always requires co-infection with a replication-competent helper virus.

Further strategies to engineered VLP of viral symbionts of parasites could, of course, be explored to improve gene delivery to different parasites, as exemplified by the use of engineered bacteriophage T4 expressing antigens facilitating specific cell targeting [120].

## Conclusion

The endosymbiont viruses of protozoan parasites are a relatively new but rapidly expanding field of research. However, when compared to the use of bacteriophage and oncolytic viruses more pure and applied research will be needed before their use can be contemplated against protozoan parasite infections in human medicine. In accord with recent efforts to establish a platform to explore and understand the parasite microbiome through an integrated approach [158], we advocate intensification of the research in this area both from the point of view of basic biology but also in areas pertinent to infectious disease including epidemiology and the potential for disease treatment and prophylaxis. Both in vivo and in vitro infection models will be required to study virus-parasite interactions both independently and in association with the human or animal hosts of the parasites [159]. Due to their complex life cycles and culture requirements, for many protozoan parasites such models are still inadequate, incomplete, since they do not replicate all the parasite life stages, do not exist, or might simply not be suited to evaluating virus behaviour [160–163].

We believe that the time is right to begin a more thorough investigation into viruses of protozoan parasites with a view to evaluating them as agents for controlling the many largely intractable infections that they produce.

## Data Availability

Not applicable.

## Abbreviations

GBDS: Global Burden of Disease Studies
PPDs: Protozoan parasitic diseases
AMR: Antimicrobial resistance
VLPs: Virus-like particles
dsRNA: Double-strand RNA
ssRNA: Single-strand RNA
PV: Parasitophorous vacuole
GLV: *Giardia lamblia* Virus
CP: Capsid protein
RdRp: RNA-dependent RNA polymerase
TVV: *Trichomonas vaginalis* Virus
LRV: *Leishmaniavirus*
LmarLBV1: *Leishmania martiniquensis* Leishbunyaviruses 1
CspV: *Cryspovirus*
MaRNAV-1: Matryoshka RNA virus 1
LepsyNLV1: *Leptomonas seymouri* Narna-like virus
